# Examining the affordable connectivity program and telehealth use: a pilot survey of the affordable connectivity program, telehealth, video and audio visits in a racially diverse, lower-income population

**DOI:** 10.1038/s41598-025-86728-y

**Published:** 2025-01-17

**Authors:** Omolola E. Adepoju, Patrick Dang, Carlos Fuentes, Winston Liaw

**Affiliations:** 1 Humana Integrated Health Systems Sciences Institute, University of Houston, Houston, USA; 2 Department of Health Systems and Population Health Sciences, Tilman J Fertitta Family College of Medicine, University of Houston, Houston, TX 77204 USA

**Keywords:** Lifestyle modification, Patient education

## Abstract

The Affordable Connectivity Program (ACP) aimed to narrow the digital divide by providing discounted internet services for millions of low-income households during the COVID-19 pandemic. This study examined associations between enrollment in the ACP and Telehealth visits in a racially diverse low-income population. Data were obtained via a cross-sectional survey of 213 respondents. Three multivariable regression models examined associations between ACP and three dependent variables, separately: (i) Used telehealth in the past 12 months, (ii) Had 1 or more video visits/consults in the past 12 months, and (iii) Had 1 or more telephone visits/consults in the past 12 months. 41% of survey respondents identified as non-Hispanic Black individuals, 33% as non-Hispanic White individuals, and 22% as Hispanic individuals. 69% reported a pre-tax annual household income of less than $35,000. Only 2 of 10 respondents had heard of ACP and were enrolled, while approximately 4 in 10 had never heard of it. Respondent knowledge/enrollment in the ACP was not significantly associated with telehealth, video consult, or telephone usage. Other demographic characteristics including race, income, educational attainment, and biological sex were significantly associated with telehealth, video, and telephone consults. We observed no association between ACP and telehealth use. While this finding is concerning, it offers an opportunity to reflect on potential reasons for ACP adoption gaps, such as digital literacy, device availability, and potential misconceptions about telehealth services.

**Supplementary Information:**

The online version contains supplementary material available at 10.1038/s41598-025-86728-y.

## Introduction

Telehealth and related technologies have significantly altered medical care following the proliferation of such technologies during the COVID-19 pandemic^1^. Others have described this transition towards telehealth as a ‘tectonic shift’ that is unlikely to be reversed^2^. Notwithstanding, technology is not without its challenges as unfamiliar interfaces may prove challenging and unintentionally prevent patients from engaging with providers^1^. This is especially true for medically underserved communities who are often at risk for sub-optimal health outcomes. These concerns, along with the magnification of the digital divide that the pandemic brought^3^, led to the initial establishment of the Consolidated Appropriation Act and Emergency Broadband Connectivity Fund in December 2020, a $3.2 billion fund that helped households afford internet services during the pandemic. This was later replaced with the Affordable Connectivity Program (ACP) in November 2021, a $14 billion program, which widened household eligibility and provided qualifying households with a discount of up for $30 per month towards internet services and a one-time discount of $100 towards a device^4^.

The ACP helped 44% of 52 million eligible households (as of January 2024) gain access to online internet services and devices^4,5^. Coordinating efforts with roughly 1,700 internet service providers and 228 grants with community partners^4^, the program worked to distribute over 8 million devices to eligible households^6^. An analysis of the 2022 American Community Survey highlights the ACP’s nuanced role in broadband adoption^7^. In the observed counties, places that experienced strong broadband adoption growth also had elevated rates of ACP enrollment. The study also observed that broadband adoption growth was the greatest in high-poverty cities from 2019 to 2021, and that ACP enrollment played a supportive role in broadband adoption growth from 2021 to 2022^7^. Additional work has shown that ACP utilization is regionally diverse, with high enrollment rates in both urban and rural congressional districts^5^.

Framing the relationship between the ACP program and telehealth utilization within the Andersen^8^ provides an understanding of how the ACP may influence health care access by acting as an enabling resource. Within this model, access to health care is determined by predisposing factors (e.g., demographics, social structure), enabling factors (e.g., resources available to individuals and communities), and need factors (e.g., perceived and actual health care needs). The ACP can be understood as an enabler by reducing a critical barrier—limited broadband access—that disproportionately affects underserved populations. By subsidizing internet services and providing affordable devices, the ACP empowers individuals to connect with telehealth platforms and e-health services that would otherwise remain inaccessible. This expanded connectivity facilitates interactions with health care providers, medication management through online portals, and engagement with remote health monitoring tools. By improving digital access, the ACP has the potential to reduce the digital divide and mitigate disparities in health care utilization, particularly among medically underserved populations.

However, evidence of the ACP on health-related measurements, including access to health care, remains limited. Although few studies have acknowledged the ACP’s role in expanding internet access, a 2023 literature synthesis by Lyles et al. highlighted that despite the rise of the ACP that there are no current structural or policy mechanisms linking internet service provision with health initiatives or delivery services, including remote monitoring^9^. Given the temporary nature of the program and depletion of funds, enrollment has set to end in February 2024, despite internal requests for additional funding to reduce consumer confusion and stabilize the number of households affected by the end of this program^4^.

This brief report examines the association between ACP enrollment and telehealth usage in a racially diverse, lower-income population. By directly touting their ability to increase telehealth utilization and other e-health services in a post-pandemic climate geared towards digitization, these programs can increase adoption rates and eventually play a significant role in decreasing disparities in telehealth utilization.

## Methods

Using purposive sampling, a cross-sectional survey was deployed among adults residing in low-income communities in Houston (Third Ward, East End), New York (Bronx, Brooklyn, Queens), and Los Angeles (East Los Angeles, Hyde Park, Huntington Park) from April to August 2023. These three areas, spanning diverse geographical locations, rank among the top five largest metropolitan areas in the United States. The inclusion criteria were i) residing in one of the target communities, and ii) having an annual household income of $50,000 or less (for couples), or $35,000 or less (single person household). The survey focused on knowledge and enrollment in the ACP and respondents’ use of telehealth and related technologies. To ensure inclusivity for individuals without technological access, the research team collaborated with local community-based organizations to distribute paper surveys in the target communities. The survey was offered in English language only.

The survey included previously published questions, drawing from the Pew Research Center, the National Health Interview Survey, the Health Information National Trends Surveys, and other published work on this topic^10^. Respondents spent on average 8 min to complete the survey, and anonymized responses were used to generate the study dataset.

Three telehealth-related measures were examined based on responses to the following questions: (i) Have you used telemedicine or telehealth technology in the past 12 months; (ii) Please indicate the number of times that you have had live video consults with healthcare provider (e.g. physician, physician assistant), in the past 12 months; (iii) Please indicate the number of times that you have had telephone consults with healthcare provider (e.g. physician, physician assistant), in the past 12 months. Responses to these three questions were used to create binary flags depicting three respective dependent variables: (i) Used telehealth in the past 12 months, (ii) Had 1 or more video visits/consults in the past 12 month, and (iii) Had 1 or more telephone visits/consults in the past 12 months.

Over 1000 surveys were disseminated, with 305 surveys being returned. Among these, 213 were fully completed and utilized for this study. We used univariate statistics to describe respondent characteristics. Chi-square tests were used to assess independent bivariate associations between survey respondent characteristics and each of the three telehealth-related measures. Three logistic regression models assessed the strength of the relationships, controlling for patient characteristics. Authors only had access to anonymized data, hence, the ethics committee at the University of Houston waived the requirement for informed consent. All research methods were performed in accordance with relevant guidelines/regulations. All data management and analyses were performed using Stata 16. This study was approved by the University of Houston institutional review board in April 2023 (Study ID- STUDY00004168).

## Results

Table 1 shows the summary characteristics of the analytic sample. Overall, 41% of survey respondents identified as Black, 33% as White, 22% as Hispanic, and less than 4% identified as Middle Eastern/Asian/Native American/Pacific Islander/Other. About half of the sample were aged 40–64 years, and 69% reported a pre-tax annual household income of less than $35,000. The sample comprised of 56% females, and with regard to highest education completed, only 20% reported having a bachelor’s degree or more. Among respondents, a majority (58.2%) reported not using telehealth in the past 12 months, with 44.6% having 1 or more video visits and 55.8% having 1 or more telephone visits in the past 12 months. Importantly, only 20.7% of respondents heard of the ACP and were enrolled, while 38.0% never heard of the program.

**Table 1:** Characteristics of survey respondents (*n* = 213).

Characteristics	Total sample
Race/Ethnicity	
White	71 (33.5)
Black	86 (40.6)
Hispanic	47 (22.2)
Other	8 (3.8)
Age group	
18–39	59 (27.7)
40–64	106 (49.8)
65+	48 (22.5)
Income	
≤ $35,000	146 (68.9)
$35,001+	66 (31.1)
Education	
High school or less	95 (43.6)
Technical/Vocational, Some college	76 (35.7)
Bachelor’s degree or more	44 (20.7)
Sex	
Male	93 (43.6)
Female	120 (56.3)
Used telehealth in the past 12 months	
No	124 (58.2)
Yes	89 (41.8)
Had 1 or more video visits/consults in the past 12 months	
No	118 (55.4)
Yes	95 (44.6)
Had 1 or more telephone visits/consults in the past 12 months	
No	94 (44.1)
Yes	119 (55.8)
Heard of the Affordable Connectivity Program	
Yes, and I am enrolled	44 (20.7)
Yes, but I am not enrolled	88 (41.3)
No, never heard of it	81 (38.0)

### Bivariate associations

Bivariate associations of independent variables by telehealth, video consult, and telephone consult usage are shown in Table 2. Telehealth usage was independently associated with race/ethnicity (53.5% White used telehealth vs. 45.4% Black, 19.2% Hispanic, 37.5% Other; *p* ≤ 0.01), income (60.6% making $35000 or more vs. 32.9% making ≤$35000; *p* ≤ 0.01), education (61.4% bachelor’s degree or more vs. 35.5% with high school or less, 38.2% technical/vocational or some college, *p* = 0.01), and sex (50.8% female vs. 30.1% male; *p* ≤ 0.01). Video consult users was also independently associated with race/ethnicity (62.5% Other used telehealth vs. 57.8% White, 34.9% Black, 40.4% Hispanic; *p* = 0.02), age (54.2% aged 18–39 years vs. 49.1% aged 40–64, 22.9% aged 65 and older; *p* ≤ 0.01), and education level (56.6% technical/vocational or some college vs. 30.1% high school or less, 54.6% bachelor’s degree or more; *p* ≤ 0.01). Telephone consult usage was not independently associated with any of the respondent characteristics.

**Table 2:** Bivariate associations between respondents’ characteristics and (a) use of telehealth, (b) video consults, and (c) telephone consults.

	Used telehealth in the past 12 months		Had 1 or more video visits/consults in the past 12 months		Had 1 or more telephone visits/consults in the past 12 months	
	Yes (n,%)	p-value	Yes (n,%)	p-value	Yes (n,%)	p-value
Race/Ethnicity		0.00		0.02		0.32
White	38 (53.5)		41 (57.8)		45 (63.4)	
Black	39 (45.4)		30 (34.9)		44 (51.2)	
Hispanic	9 (19.2)		19 (40.4)		26 (55.3)	
Other	3 (37.5)		5 (62.5)		3 (37.5)	
Age group		0.43		0.00		0.58
18–39	22 (37.3)		32 (54.2)		31 (52.5)	
40–64	49 (46.2)		52 (49.1)		63 (59.4)	
65+	18 (37.5)		11 (22.9)		25 (52.1)	
Income		0.00		0.16		0.06
≤ $35,000	48 (32.9)		60 (41.1)		75 (51.4)	
$35,001+	40 (60.6)		34 (51.5)		43 (65.2)	
Education		0.01		0.00		0.48
High school or less	33 (35.5)		28 (30.1)		49 (52.7)	
Technical/Vocational, Some college	29 (38.2)		43 (56.6)		42 (55.3)	
Bachelor’s degree or more	27 (61.4)		24 (54.6)		28 (63.6)	
Sex		0.00		0.21		0.27
Male	28 (30.1)		37 (39.8)		48 (51.6)	
Female	61 (50.8)		58 (48.3)		71 (59.2)	
Heard of Affordable connectivity program		0.06		0.07		0.65
Yes, and I am enrolled	17 (38.6)		14 (31.8)		25 (56.8)	
Yes, but I am not enrolled	30 (34.1)		38 (43.2)		46 (52.3)	
No, never heard of it	42 (51.9)		43 (53.1)		48 (59.3)	
Overall % responding yes to (a) use of telehealth, (b) video consults, and (c) telephone consults.	47 (52.8)		52 (54.7)		71 (59.7)	

### Multivariate associations

Three multivariable regression models examined factors associated with telehealth, video consult, and telephone consult usage in Table 3. The telehealth model found that compared to white respondents, Black and Hispanic respondents were significantly less likely to have used telehealth (OR: 0.40, *p* = 0.02; OR: 0.16; *p* ≤ 0.01). Respondents making higher income were also more likely to use telehealth when compared to those making less than $35,001 (OR: 3.59, *p* = 0.01), along with female respondents (OR: 2.32, *p* = 0.01). In the video visit model, Black and Hispanic respondents were also less likely to use video visits compared to their white counterparts (OR: 0.23, *p* ≤ 0.01; OR: 0.31, *p* = 0.01), along with older age (age 65 or older OR: 0.14, *p* ≤ 0.01). Those with technical/vocational or some college education were significantly more likely to have video visits compared to their counterparts with a high school education or less (OR: 4.60, *p* ≤ 0.01). For the telephone visit model, black respondents were less likely to use telephone visits compared to white respondents (OR: 0.39, *p* = 0.01), while higher income was greater associated with telephone visits than their counterparts ($35,001 + OR: 2.62, *p* = 0.03). Respondent knowledge/enrollment in the ACP were not significantly associated with telehealth, video consult, or telephone usage.

**Table 3:** Multivariable regression models for (a) use of telehealth, (b) video consults, and (c) telephone consults.

	Used telehealth in the past 12 months		Had 1 or more video visits/consults in the past 12 months		Had 1 or more telephone visits/consults in the past 12 months	
	OR	p-value	OR	p-value	OR	p-value
Race/Ethnicity						
White	Ref		Ref		Ref	
Black	0.40	0.02	0.23	0.00	0.39	0.01
Hispanic	0.16	0.00	0.31	0.01	0.73	0.45
Other	0.23	0.09	0.46	0.37	0.22	0.06
Age group						
18–39	Ref		Ref		Ref	
40–64	0.90	0.79	0.66	0.27	1.32	0.43
65+	0.52	0.17	0.14	0.00	0.97	0.95
Income						
≤ $35,000	Ref		Ref		Ref	
$35,001+	3.59	0.01	1.34	0.54	2.62	0.03
Education						
High School or less	Ref		Ref		Ref	
Technical/Vocational, Some college	1.09	0.81	4.60	0.00	1.17	0.64
Bachelor’s degree or more	1.02	0.97	2.47	0.10	1.04	0.93
Sex						
Male	Ref		Ref		Ref	
Female	2.32	0.01	1.60	0.15	1.33	0.33
Heard of affordable connectivity program						
Yes, and I am enrolled	Ref		Ref		Ref	
Yes, but I am not enrolled	0.47	0.09	0.63	0.32	0.58	0.19
No, never heard of it	0.74	0.53	0.98	0.87	0.58	0.24

## Discussion

In this pilot study, we found that only 2 of 10 respondents had heard of ACP and were enrolled, while approximately 4 in 10 had never heard of it. Importantly, we observed no association between ACP and telehealth use. While this finding is concerning, it offers an opportunity to reflect on potential reasons for ACP adoption gaps. Our finding that few people were aware and enrolled in the program suggests that ongoing outreach and communication strategies may not be effectively reaching the target audience. Additionally, individuals who might have benefited from the program may have faced challenges such as limited digital literacy or language barriers, which could have impeded their ability to participate effectively.

Considering that device affordability and availability are crucial factors, even with subsidized connectivity, individuals may lack the necessary devices (such as smartphones, tablets, or computers) to access telehealth services. Finally, we posit that there might be misconceptions that discourage individuals from participating, such as concerns about data privacy, security, or the overall purpose of the program.

Notably, certain participants in this study might not have possessed a clear comprehension of telehealth. Despite stating that they engaged in video or telephone consultations with their healthcare provider, these respondents did not perceive these interactions as constituting telehealth. This contributes to the lower affirmative response rate on telehealth use, compared to video consults or telephone consults in this study. In addition, affirmative responses to telehealth use in this study were seemingly higher, compared to other studies in low-income populations that reported telehealth rates of 20–30%^11^.

Controlling for ACP, racial and ethnic minorities were less likely to have used telehealth, video visits or telephone visits, compared to their white counterparts. This finding aligns with earlier work, for example, during the peri-pandemic period, researchers noted that individuals adopting telemedicine for the first time tended to be of white ethnicity, younger age, and higher socioeconomic status compared to those who did not utilize telemedicine^12,13^. This digital divide is attributed to socioeconomic factors, especially in lower-income minoritized populations who are more likely to report lower rates of smartphone ownership (71% compared to 85% among Caucasians)^14^, reliable internet access^15^ (50% compared to 80% among Caucasians)^16^, and digital literacy^15^ (53% compared to 89% among Caucasians)^17^. This gap is furthered by the fact that Black and Latinx patients are overrepresented in low-paying essential industries^18^, where video visits during work may not be convenient. These groups may also report challenges obtaining reliable and consistent internet access^19^. We posit that telephone visits may not actually be explicitly preferred, but are the only option for low-income and racial patients seeking to engage in telemedicine^20^.

Our finding that older adults are significantly less likely to have video visits also aligns with earlier studies comparing audio only versus video visits. Work by Pasquinelli et al. highlighted that among older adults aged 50+, telephone visits were preferred^21^ Rodriquez and colleagues^22^ in their examination of the differences in the use of telephone and video telemedicine visits during the COVID-19 pandemic noted that older adults were less likely to have video visits, with 17% completing telephone visits versus 11% video visits. This brief report also found that earning $35,001 or more was associated with an increased odds of using technology modalities, aligning with earlier studies. For example, among those with a median household income of under $50,000, 29% completed telephone versus 16% video visits^13^. Work by Hsaio aligns with this earlier trend associating lower video visit use with those who are self-pay/uninsured, living in rural areas, and non-English speaking^23^.

Females, and having more than a high school degree were both strongly associated with telehealth and video visits. These findings are in alignment with work examining both telehealth^24^ and video visits^25^. Additionally, being female^26^ and having a college degree^27^ have also been found to be significantly associated with higher e-Health literacy (eHL) rates. Given the role that eHL and video capability play in understanding telemedicine, both characteristics play a significant role in improving likelihood of video visits^27^ and overall telehealth utilization.

There are many limitations to be considered. First, this is a relatively small sample, which may contribute to our inability to detect significant findings. Our data were collected from only three cities by community partners who used a convenience sampling approach to target patients who met the eligibility criteria—this can potentially restrict the generalizability of our findings to broader populations. Future studies should expand the geographic scope to include more diverse locations to improve external validity. Second, some respondents may have not had a clear understanding of telehealth. While some indicated they had video or telephone visits, they did not consider these visits as telehealth. Third, we did not control health status or insurance, and considering the role that both measures play in healthcare access, this is an area that deserves further exploration. Fourth, recall or knowledge bias may influence responses, as some participants might have utilized the ACP subsidy without realizing it was specifically part of the ACP program. Finally, the survey was only available in English, which can be a significant limitation in generalizing to Spanish-speaking populations. Future iterations of this research should incorporate multilingual options to ensure inclusivity and improved generalizability.

In conclusion, this pilot study highlights a gap between awareness and enrollment in the ACP and its potential impact on telehealth utilization among a racially diverse, low-income population. Despite the ACP’s intent to bridge the digital divide, the lack of significant association between ACP enrollment and telehealth usage suggests that broader systemic barriers—such as digital literacy, access to appropriate devices, and misconceptions about telehealth—may hinder its effectiveness. These preliminary findings underscore the need for targeted outreach efforts, education, and structural interventions to improve awareness and accessibility of programs like the ACP. Addressing these barriers is critical to ensuring that such initiatives achieve their intended goal of promoting equitable access to telehealth and reducing disparities in digital health utilization.

## Electronic supplementary material

Below is the link to the electronic supplementary material.

Supplementary Material 1

## Data Availability

The datasets used and/or analyzed during the current study are available from the corresponding author on reasonable request, in line with IRB guidelines.
