# Supplementary material for: Examining the affordable connectivity program and telehealth use: a pilot survey of the affordable connectivity program, telehealth, video and audio visits in a racially diverse, lower-income population
Source: Sci Rep. 2025 Jan 17;15:2321. doi: 10.1038/s41598-025-86728-y (PMC11747251; doi:10.1038/s41598-025-86728-y)
Supplement: Supplementary file 1 — Supplementary Material 1 [file 41598_2025_86728_MOESM1_ESM.docx]

Technology Usage and Knowledge Survey Questions (Qualtrics)

**Please tell us about yourself**

We would like to ask you some questions about yourself. If you prefer not to answer a particular question, simply leave the answer blank and move on to the next question.

**Please select your age group:**

- 18-29
- 30-39
- 40-49
- 50-64
- 65+

**With regard to gender, what do you identify as?**

- Male
- Female
- Prefer to self-describe: ______________

**What is your marital status?**

- Single
- Married
- Divorced
- Separated
- Widowed

**How many people live with you now (including yourself)? _____**

**What is the highest degree or level of education you have completed?**

- Never received high school diploma/GED
- High school diploma/GED
- Technical/vocational degree
- Some college level credits or 2-year college degree
- Bachelor’s degree
- Master’s degree
- PhD or other professional degree

**Check all of the following categories that describe your race/ethnicity.**

- Arab / Middle Eastern
- Asian / Asian American
- Black / African American
- Hispanic / Latina/o/x
- Native American / American Indian / First Nation
- Pacific Islander / Native Hawaiian
- White / Caucasian
- Other (Please Specify: ____________)

**What is your annual household income before taxes?**

- Less than $25,000
- $25,000 - $34,999
- $35,000 - $49,999
- $50,000 - $74,999
- $75,000 - $99,999
- $100,00 - $149,999
- $150,000 or more
- Unemployed

**What is your zip code? _________________**

**What kind of health insurance do you have?**

- Medicare
- Medicaid
- TRICARE/Military/VA/CHAMP
- Private health insurance
- I do not have health insurance

**Available Technology**

We would like to know about your available technology and experiences. If you prefer not to answer a particular question, simply leave the answer blank and move on to the next question.

**Do you subscribe to dial-up internet service at home... OR do you subscribe to a higher-speed broadband service such as DSL, cable, or fiber optic service?**

- Dial-up
- Higher-speed
- Both Dial-up and Higher-speed
- Access Internet on Smartphone or Tablet only
- I do not have internet service at home
- Not sure

[code] If previous question is Dial-up, following question is presented:

**Just to confirm, you use a dial-up connection to the internet at home, and not a higher-speed broadband connection?**

- Yes
- No

**Do you have a cable or satellite television subscription?**

- Yes
- No

**Do you own a cell phone?**

- Yes
- No

[code] If previous question is yes, following question is presented:

**Is your cell phone a smartphone?**

**A smartphone has some advanced features, such as web browsing and software applications (Social Media/Games)**

- Yes
- No

**Do you own a tablet with access to the internet?**

- Yes
- No

**Do you own a computer or have access to one?**

- Yes, it is a personal one
- Yes, I have a loaner laptop or visit local organizations with public computers
- No

**Have you heard of the Affordable Connectivity Program (ACP)?**

- Yes, I am enrolled in the program
- Yes, I have heard of the program but am not enrolled
- No, I have not heard of the program

**Technology & Healthcare**

We would like to know about your personal technology usage, online health behaviors and experiences when searching for healthcare information. If you prefer not to answer a particular question, simply leave the answer blank and move on to the next question.

**Have you used telemedicine or telehealth technology in the past 12 months?**

- Yes, I have used telemedicine/telehealth technology
- No, but I have heard about telemedicine/telehealth technology
- No, I have not used telemedicine/telehealth technology

**Have you ever accessed the internet on a computer/tablet/smartphone for any of the following in the past 12 months:**

Look for health information for self-diagnosis _Yes_No

Look for health information to better understand medical diagnosis _Yes_No

Fill a prescription _Yes_No

Schedule an appointment with a health care provider _Yes_No

Communicate with a health care provider by email _Yes_No

View electronic health records and medical notes _Yes_No

**Based on the results of your most recent search on the internet for information about a health concern, how much do you agree or disagree with each of the following statements?**

It took a lot of effort to get the information I needed

- Strongly disagree
- Somewhat disagree
- Somewhat agree
- Strongly agree

I easily understood the health information I obtained

- Strongly disagree
- Somewhat disagree
- Somewhat agree
- Strongly agree

I was concerned about the quality of the information

- Strongly disagree
- Somewhat disagree
- Somewhat agree
- Strongly agree

**Based on your previous internet experiences, how much do you agree or disagree with each of the following statements?**

I use information form the internet to make decisions about my health

- Strongly disagree
- Somewhat disagree
- Somewhat agree
- Strongly agree

I use information from the internet in discussions with my health care provider

- Strongly disagree
- Somewhat disagree
- Somewhat agree
- Strongly agree

I find it hard to tell whether health information on the internet is true or false

- Strongly disagree
- Somewhat disagree
- Somewhat agree
- Strongly agree

I’m afraid my personal health information will be misused

- Strongly disagree
- Somewhat disagree
- Somewhat agree
- Strongly agree

**Have you used an electronic wearable device to monitor or track your health or activity? For example, a Fitbit, Apple Watch, or Garmin Vivofit.**

- Yes, I wear one regularly
- Yes, but I no longer wear one
- No, I have never used one

**Have you used apps or computer programs from the medical field, e.g. as a calorie counter, medication reminder, blood sugar documentation, pain diary, etc.?**

- Yes, I use them often
- Yes, but I no longer use them
- No, I have never used one

**Please identify the average number of times that you have participated in the following in the past 12 months**

|  | 0 | 1 | 2 | 3 | 4 | 5 or more |
| --- | --- | --- | --- | --- | --- | --- |
| Telephone consults with healthcare provider (eg physician, physician assistant) |  |  |  |  |  |  |
| Live video consults with healthcare provider (eg physician, physician assistant) |  |  |  |  |  |  |
| Remote patient monitoring (RPM)* |  |  |  |  |  |  |
| Other (please specify_____) |  |  |  |  |  |  |

*RPM: technology to remotely monitor patients’ health data and deliver it to providers for additional review and action

END

If you would like to learn more about the Affordable Connectivity Program, including eligibility and how to enroll, visit [www.affordableconnectivity.gov/](http://www.affordableconnectivity.gov/) after the completion of the survey.
